# Efficient joint noise removal and multi exposure fusion

**DOI:** 10.1371/journal.pone.0265464

**Published:** 2022-03-25

**Authors:** Antoni Buades, Jose Luis Lisani, Onofre Martorell

**Affiliations:** Institute of Applied Computing and Community Code (IAC3) and with the Dept. of Mathematics and Computer Science, Universitat de les Illes Balears, Palma, Spain; University of Engineering & Technology, Taxila, PAKISTAN

## Abstract

Multi-exposure fusion (MEF) is a technique that combines different snapshots of the same scene, captured with different exposure times, into a single image. This combination process (also known as fusion) is performed in such a way that the parts with better exposure of each input image have a stronger influence. Therefore, in the result image all areas are well exposed. In this paper, we propose a new method that performs MEF and noise removal. Rather than denoising each input image individually and then fusing the obtained results, the proposed strategy jointly performs fusion and denoising in the Discrete Cosinus Transform (DCT) domain, which leads to a very efficient algorithm. The method takes advantage of spatio-temporal patch selection and collaborative 3D thresholding. Several experiments show that the obtained results are significantly superior to the existing state of the art.

## 1 Introduction

Multi-exposure fusion (MEF) methods combine different pictures of the same scene, captured with different exposure times, into a single image. By keeping the best exposed parts of each image, it is possible to reconstruct a result where all the details of the scene are well rendered. Compared to High Dynamic Range (HDR), MEF doesn’t require the estimation of the camera response function and the tone mapping of the processed values to the standard 8-bit [0, 255] range.

The differences between the existing MEF algorithms in the literature lie in the way the information of the input images is blended. The classical method by Mertens et al. [1] computes weighted averages of the values of the input images at the same pixel location, the weights depending on exposure, saturation and contrast. These averages can also be computed in the Discrete Cosinus Transform (DCT) domain [2]. Other authors [3, 4] use gradient information, and obtain the final result by solving a Poisson partial differential equation. Moreover, to prevent the appearance of visual artifacts in the resulting images, the blending is not applied on a per-pixel basis. Instead, a patch-based approach or a multiresolution blending strategy are used [5–7].

The use of image fusion techniques is not limited to multi-exposure images. Indeed, the combination of several images permits to improve their quality, removing for example noise [8], compression artifacts [9], haze [10–12], blur [13] or shaking blur from hand held video [14, 15].

In this paper we tackle the problem of fusing noisy multi-exposed images. A naive approach would consist in independently denoising the multi-exposed images and then applying a fusion technique to the result. We address both problems jointly for the first time in the literature, to the best of our knowledge. We develop a technique that draws inspiration from the well known BM3D denoising method [16] and the multi-exposure fusion method described in [2].

BM3D denoising takes advantage of the redundancy of similar image patches (i.e. small image square blocks). Each image patch is denoised by grouping together similar patches in a local neighborhood and stacking them in a 3D structure to which a 3D transform is applied. In practice, the 3D transform is applied in a separable way, i.e. 2D DCT transforms followed by a 1D DCT or Walsh-Hadamard transform, as detailed in [16]. Denoising is achieved by applying a shrinkage operator to the coefficients in the transformed domain. This denoising technique is known as collaborative filtering.

We adapt BM3D to denoise the whole set of multi-exposure images by using the spatio-temporal patch selection strategy proposed in [8]. The fusion method in [2], on the other hand, merges the 2D DCT coefficients of differently exposed images. This method is not able to merge the coefficients describing the average of the patch and copies this value from the Mertens et al. [6] solution. In this paper we modify the algorithm to get rid of the dependence on Mertens et al.’s result, and include an additional step to make it robust to noise. The fact that both BM3D and the proposed fusion method work on the 2D DCT domain permits to combine them to obtain an efficient algorithm that simultaneously denoises and fuses the multi-exposed images.

**Fig 1:**
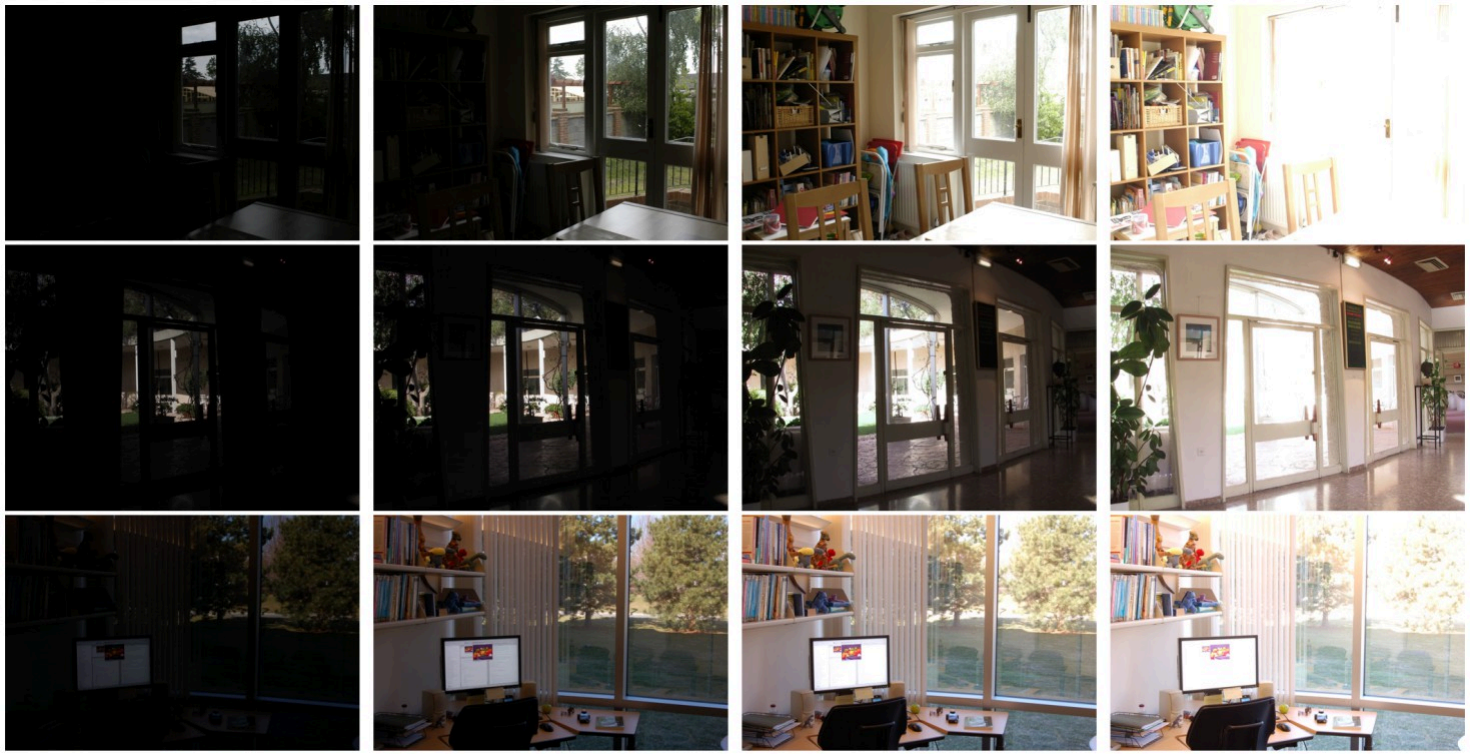
Multi exposure data sets used for comparison.

The performed experiments in the sequences of Fig 1 show the superior performance of the proposed technique over the naive approach, both in terms of quality of the results and computational efficiency. The paper is organized as follows: Section 2 describes the existing literature on MEF methods. The fusion method is described in Section 3. Section 4 presents the complete proposed technique, including the BM3D-inspired denoising stage and the fusion method. In Section 5, we discuss the implementation of the method and compare with state of the art algorithms. Finally we draw some conclusions in Section 6.

## 2 Related work

The literature on MEF is very extensive. Mertens et al. [1] proposed to combine the images by averaging, choosing for each pixel a different weight depending on saturation, contrast and well-exposedness. Since such an average produces ghosting effect if the images are not well aligned, several methods were proposed to take motion into account, such as An et al. [17], Liu et al. [18], Hessel et al. [19] Li et al. [20], Ocampo et al. [21] and Hayat et al. [22].

Instead of averaging directly pixel values, several methods prefer to fuse gradient information, and then obtain the final image by solving a Poisson equation [3]. Some authors [4, 23–25] propose to merge all gradient values, while others (Kuk et al. [26]) choose the gradient corresponding to the better exposed image.

Deciding independently for each pixel which is the correct combination is not a robust strategy and leads to visual artifacts. Common approaches to improve the results involve the use of pyramidal image representations or patches. Several methods [6, 7, 27, 28] adopted the Laplacian pyramid [29]. Patch-based methods make the fusion more robust by involving all the pixels in a small window [5, 30–32]. The method in [2] uses a DCT transform. The 2D DCT coefficients of the patches at the same spatial location and different exposure are combined depending on its magnitude. This combination is not valid to set the patch illumination, which is obtained using the Mertens et al. [6] algorithm.

The use of alternative color systems to the standard RGB is often proposed, specially YCbCR [33] which separates the luminance from the chromatic components. Since the chromatic components contain few high frequency information, a simpler strategy can be used to fuse them. Published methods differ depending on which one of the previously exposed techniques is applied to the Y component [2, 34, 35].

For non static sequences, the fusion creates ghosting effects near the boundary of moving objects. A possible strategy to address this problem is to weight the fusion considering patch correlation, thus discarding dissimilar patches which might belong to a different object. Another common choice is to register the whole sequence into a reference exposure [2, 36–38]. A straightforward strategy is to select the better exposed image as reference and generate a new sequence where the color of each image is equalized with respect to this reference. This new sequence can be fused using a MEF method, as proposed in [39–42]. Such a strategy avoids the creation of ghosting effects due to motion, but the fusion does not permit to get rid of noise.

Several methods (Z. G. Li et al. [43, 44], Singh et al. [45], Raman et al. [46], Li et al. [47]) divide the images into low and high frequency components using the Bilateral [48] or the Guided filter [49]. This separation permits an additional enhancement of the image details.

Multi-exposure fusion might be accomplished by the use of variational techniques [50–52]. The proposed methods favor the geometry of the short exposure views and the chromaticity of the ones with longer exposure. Other methods are defined in order to maximize particular quality measures as [53–56].

Recently, neural networks have been proposed for multi exposure fusion. They can be divided into two categories, depending on whether they require the ground truth to be correctly exposed in all image areas to define the loss function (supervised methods) or not (unsupervised or self-supervised method). For HDR Kalantari et al. [57] and Wu et al. [58] proposed supervised methods. For MEF, Xu et al. [59] proposed a Generative Adversarial Network (GAN) strategy to fuse pairs of images with different exposure time. In the group of the self-supervised methods, Prabhakar et al. [60, 61] designs a metric which evaluates the quality of the multi-exposure fusion image. Xu et al. [62] trained a self-supervised neural network to preserve the similarity between the fusion result and the source exposures.

Li et al. (CNNFEAT) [63] combines the several exposures depending on a series of descriptors learnt by a neural network. Zhang et al. [64] and Zhang et al. [65] propose unified deep learning frameworks for several fusion tasks, including MEF.

There is very few literature dealing with noise removal during multi-exposure image fusion, and most published papers are focused on HDR. Akyuz et al. [66] denoise each frame before fusion, but this is performed in the radiance domain. Tico et al. [39] combine an initial fusion result with the image in the sequence with the shortest exposure. This combination is performed in the wavelet domain and coefficient attenuation is applied to the coefficients of the difference. Min et al. [67] filter the set of images with spatio-temporal motion compensated anisotropic filters prior to HDR reconstruction. Lee et al. [68] use sub-band architecture for fusion, with a weighted combination using a motion indicator function to avoid ghosting effects. The low frequency bands are filtered with a multi-resolution bilateral filter while the high frequency bands are filtered by soft thresholding. Ahmad et al. [69] identify noisy pixels and reduce their weight during image fusion.

## 3 MEF algorithm

We propose a novel algorithm for multi-exposure fusion which will serve as basis for the joint fusion and denoising method. It operates in the DCT domain and it is inspired by the fusion method in [2]. Compared to [2], we introduce a new strategy for fusing the average coefficient of the DCT and a completely different color management. It also includes a novel noise removal step, allowing for its application with moderately noisy images.

The basic algorithm, applied to single-channel images, is described first. Its extension to color images is presented in Section 3.2. Finally, a modification that confers denoising capability to the method is proposed in Section 3.3.

### 3.1 Single channel image fusion

Let’s denote by *Y*_*k*_, *k* = 1, 2, …, *K* a sequence of luminance images acquired with different exposure taking values in the range [0, 1]. This sequence is supposed to be static. We split the images *Y*_*k*_ into *n*_*b*_ partially-overlapped patches of *b* × *b* pixels, $\left\{B_{k}^{l}\right\},\;l=1,\ldots ,n_{b}$, and the 2D DCT transform of each patch is computed.

At a given pixel location, some of these patches may belong to under or over exposed parts of the images, while others may be well exposed. Both under and over exposed patches will have non-zero DCT coefficients of small magnitude, due to the lack of high frequency information. Conversely, the coefficients of well exposed patches will be large. These considerations lead to the following equation that aggregates the non-zero DCT coefficients of all the patches corresponding to the same spatial location:

$$\begin{array}{c} {\hat{B}}^{l}\left(\xi \right)=\sum_{k=1}^{K}w_{k}^{l}\left(\xi \right){\hat{B}}_{k}^{l}\left(\xi \right),\;\xi \neq 0,\;l=1,2,\ldots ,n_{b}, \end{array}$$ (1)

where ${\hat{B}}_{k}^{l}$ denotes the DCT transform of patch *l* in image *k*, and the weights $w_{k}^{l}\left(\xi \right)$ are defined depending on the frequency *ξ*,

$$\begin{array}{c} w_{k}^{l}\left(\xi \right)=\frac{|{\hat{B}}_{k}^{l}{\left(\xi \right)|}^{p}}{\sum_{n=1}^{K}{\left|{\hat{B}}_{n}^{l}\left(\xi \right)\right|}^{p}}\;\xi \neq 0\;. \end{array}$$ (2)

where *p* > 0 is a parameter of the method. We observe that, for a given frequency, patches having higher coefficient magnitudes (i.e. well exposed patches) contribute more than the others to the weighted sum in Eq (1).

This strategy does not apply to the zero frequency DCT coefficient, *ξ* = 0, i.e. the average of the patch values. Since large zero frequency coefficients correspond to over-exposed images, applying the same weighted combination would simply overexpose the fused image. We weight these coefficients depending on the average value of the patch and on the average value of the image to which it belongs:

$$\begin{array}{c} {\hat{B}}^{l}\left(0\right)=\sum_{k=1}^{K}w_{k}^{l}\left(0\right){\hat{B}}_{k}^{l}\left(0\right),\;l=1,2,\ldots ,n_{b}, \end{array}$$ (3)

where

$$\begin{array}{c} w_{k}^{l}\left(0\right)=\frac{1}{C}e^{-{\left({\hat{B}}_{k}^{l}\left(0\right)-0.5\right)}^{2}/\sigma_{l}^{2}}\cdot e^{-{\left(\mu_{k}-0.5\right)}^{2}/\sigma_{g}^{2}}, \end{array}$$ (4)

with *μ*_*k*_ the average of the values of image *k*, *C* a normalizing constant, and *σ*_*l*_ and *σ*_*g*_ parameters of the method. Observe that 0.5 corresponds to the center of the [0, 1] range. Well exposed images are supposed to have an average value close to 0.5. The weighting factor (4) favors patches whose average value is close to this central value, and that belong to well exposed images. For all the experiments in this paper we fixed *σ*_*g*_ = 0.3 and *σ*_*l*_ = 0.5.

Finally, the fused patches are obtained after applying the inverse DCT transform to the result of the aggregation

$$\begin{array}{c} B^{l}\left(x\right)=F^{-1}\left(\hat{B^{l}}\left(\xi \right)\right),\;l=1,\ldots ,n_{b}. \end{array}$$ (5)

Since patches are partially overlapped, the pixels in overlapping areas are averaged to produce the final image.

### 3.2 Color image fusion

We use an orthonormal version of the well known YUV color space, described by the following linear transformation.

$$\begin{array}{c} \left(\begin{array}{c} \text{Y}\\ \text{U}\\ \text{V} \end{array})=(\begin{array}{ccc} \frac{1}{\sqrt{3}} & \frac{1}{\sqrt{3}} & \frac{1}{\sqrt{3}}\\ \frac{\sqrt{2}}{2} & 0 & -\frac{\sqrt{2}}{2}\\ \frac{1}{\sqrt{6}} & -\frac{2}{\sqrt{6}} & \frac{1}{\sqrt{6}} \end{array})(\begin{array}{c} \text{R}\\ \text{G}\\ \text{B} \end{array}\right) \end{array}$$

The Y channel, given by the normalized average of the RGB values, represents the luminance, while U and V contain the chromatic information. The use of an orthonormal transform is motivated by the denoising stage described below. The matrix rows are mutually orthogonal which guarantees that noise is not color correlated by the transform. Each row has an Euclidean norm equal to one which permits to maintain the same noise standard deviation of the original image. This transformation can be obtained from the classical YUV decomposition by applying an orthogonalization method [70]. This color transformation was used for example in [16].

**Fig 2:**
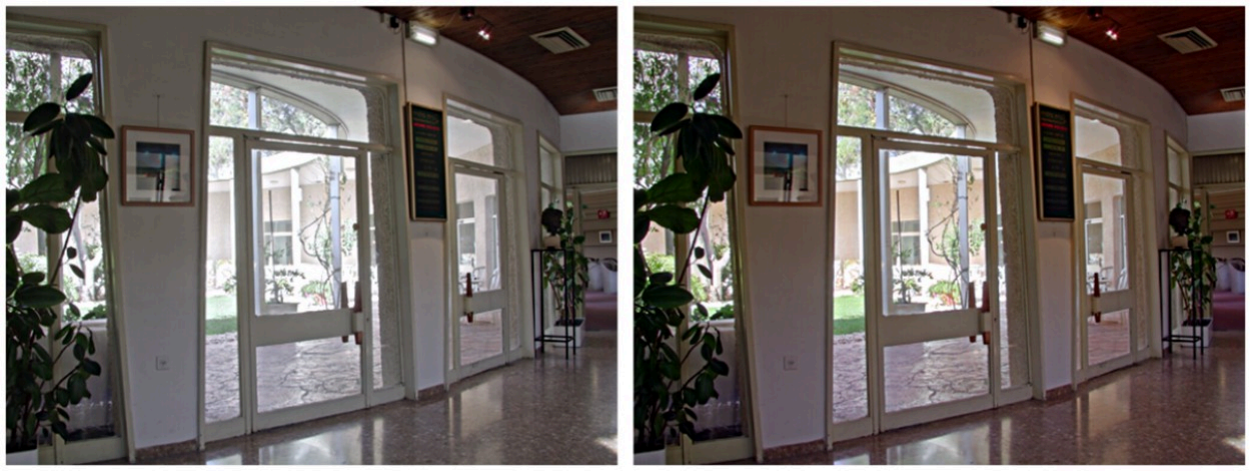
Comparison between applying the fusion to each RGB channel independently and using the YUV luminance and chromatic components. The luminance of the two results is identical, while the color is enhanced by the latter strategy.

The Y channel is processed as described in the previous section. However, for the U and V components, we apply the weighted average defined by Eqs (1) and (2) to all the coefficients, including *ξ* = 0. The reason is that aggregating the average values of the chromaticity components (i.e. the values of the zero coefficients in the transformed domain) does not increase the risk of over-exposure, but enhances the patch average chromaticity, making the result more colorful than when applying the single channel method to each of the R, G and B components. This is noticeable in Fig 2, in which both strategies are compared.

### 3.3 Noise removal

The most common noise model is the additive white Gaussian noise (AWGN). The observed noisy image *v* is related to the underlying clean image *u* by

$$\begin{array}{c} v=u+n, \end{array}$$

being *n* a noise image, independently and identically distributed at each pixel as a zero-mean Gaussian random variable with standard deviation *σ*. For other types of noise, the initial data can be modified by using variance stabilization transforms or whitening strategies, or the designed algorithm might be modified by adapting locally the parameters or applying multiscale methods. For this reason, AWGN is the most commonly assumed model in order to design general noise removal algorithms.

Denoising can be achieved by using a thresholding estimator that projects the noisy image to an orthonormal basis and reconstructs the denoised result with the transform coefficients larger than a given threshold [71].

Following this principle, the fusion method proposed in Sections 3.1 can naturally incorporate noise removal by modifying the weight definition (2) to

$$\begin{array}{c} w_{k}^{l}\left(\xi \right)=\frac{Thr_{\sigma }\left(\right|{\hat{B}}_{k}^{l}\left(\xi \right){\left|\right)}^{p}}{\sum_{n=1}^{K}Thr_{\sigma }\left(\right|{\hat{B}}_{n}^{l}\left(\xi \right){\left|\right)}^{p}},\;\xi \neq 0 \end{array}$$ (6)

where

$$Thr_{\sigma }\left(\right|{\hat{B}}_{k}^{l}\left(\xi \right)\left|\right)=\{\begin{array}{cc} 0 & |{\hat{B}}_{k}^{l}\left(\xi \right)|<T\cdot \sigma \\ |{\hat{B}}_{k}^{l}\left(\xi \right)| & \text{otherwise} \end{array}$$

**Fig 3:**
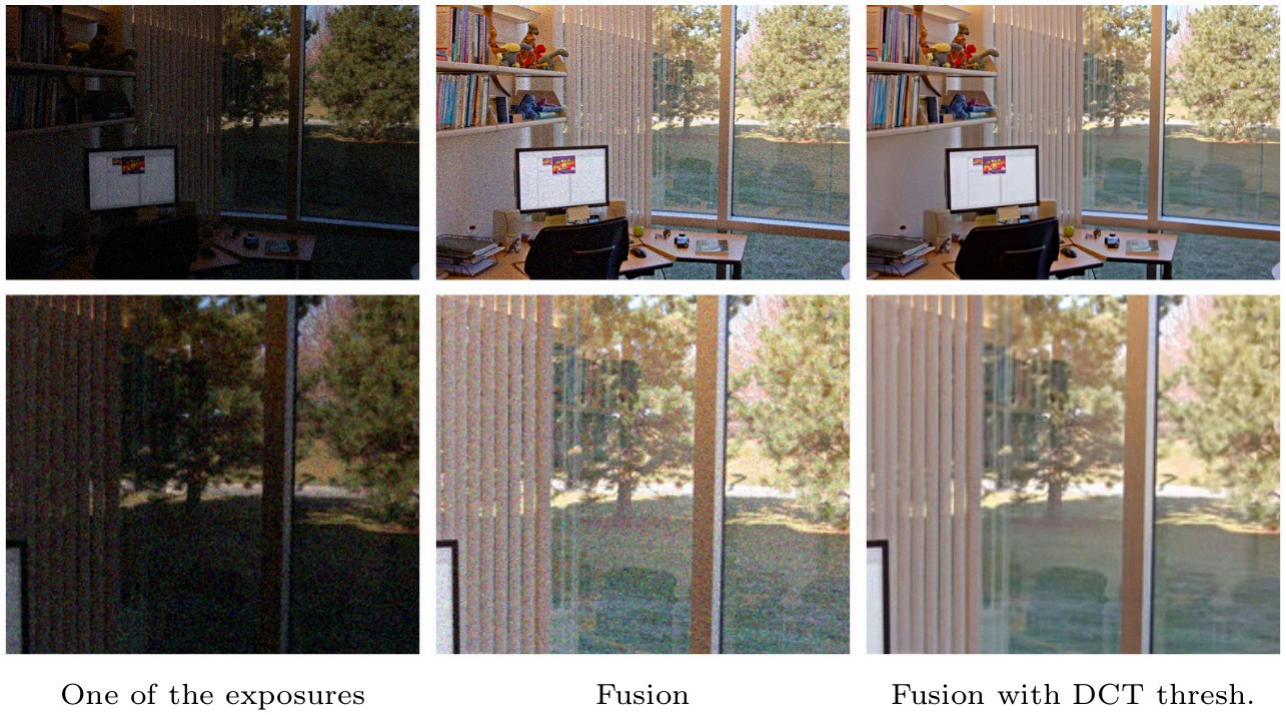
Noisy example with sequence office and noise with standard deviation *σ* = 15. Top, from left to right: one image from the multi-exposure set, fusion without DCT thresholding, fusion with DCT thresholding. Bottom: detail of the images.

Since the modified YUV color transformation is orthonormal, the noise standard deviation is not modified by the linear transformation proposed in Section 3.2, converting from RGB to the mentioned space. Thus, the same thresholding can be applied to each channel. The threshold parameter *T* is set to 2.7 as usual when denoising by thresholding in an orthonormal basis [16]. Fig 3 compares the application of the fusion chain with and without this DCT thresholding stage.

When dealing with high levels of noise, the DCT thresholding method described above is not enough to provide good denoising results. The next section describes how the fusion method can be combined with a collaborative denoising technique to obtain much better results.

## 4 Joint noise removal and fusion procedure

We assume, as in the previous sections, an AWGN noise model with standard deviation *σ* and that the input images are co-registered. Many variants have been proposed to deal with the noise removal of image sequences having the same exposure and noise conditions. Such methods cannot be directly used for multi-exposed images.

We propose a joint noise removal and fusion procedure. The noise removal stage is an adaptation of the BM3D collaborative DCT thresholding technique proposed in [16]. The use of such technique permits a natural integration with the fusion method proposed in Section 3, since both denoising and fusion are performed in the DCT domain.

BM3D is a patch-based image denoising method, which means that first the image is split into overlapping patches which are processed independently. Each patch is denoised by finding groups of similar patches in a local neighborhood and stacking them in a 3D structure. A separable 3D transform is applied to this structure (that is, 2D DCT transforms of each patch followed by a 1D transform in the third dimension of the stack) and denoising is achieved by setting to zero all the coefficients below a fixed threshold that depends on the standard deviation of the noise (which is assumed to be known). After computing the inverse 3D transform of the thresholded coefficients a denoised stack of patches is obtained. Since patches are partially overlapped the final denoised image is obtained after averaging, at each pixel position, the contributions of each denoised patch, in a process known as aggregation. This process should not be confused with the aggregation in the DCT domain described in Section 3.1 and modeled by Eqs (1) and (3).

In the original BM3D implementation this process is repeated two times, first applied to the noisy image, and then using as input both the noisy image and the first denoising result (known as oracle). In this second step the thresholding operation in the DCT domain is replaced by a Wiener filtering guided by the oracle image. The authors show that this two steps process restores more details and improves the denoising performance.

A naive solution to our problem would consist in denoising each multi-exposed image with the previous algorithm and then apply the fusion method described in Section 3 to the denoised set. However, we propose a more efficient method, which produces better results.

Instead of processing each image independently, we use information of the whole multi-exposed set to denoise each image. In particular, the group of similar patches used to create each 3D stack is searched using the patch-selection procedure proposed in [8], which has proved to reduce the dependence on noise in the patch comparison, improving the robustness of the denoising method and reducing the usual artifacts of collaborative filtering.

Given the multi-exposed set of images and an initial patch location ***x***, we associate to ***x*** a 3D block composed by the 2D patches from the set located at the same spatial position. Then, we search for similar 3D blocks associated to other spatial locations ***y***. The distance between different 3D blocks is computed as

$$\begin{array}{c} d_{\text{3D}}\left(x,y\right)=\sum_{\text{image}\;i\;\text{in}\;\text{set}}\left|\right|P_{i}\left(x\right)-P_{i}\left(y\right)\left|\right| \end{array}$$ (7)

where *P*_*i*_(***x***) and *P*_*i*_(***y***) denote the 2D patches referenced by ***x*** and ***y*** in image *i* (each 2D patch is referenced by its top-left vertex). We extract from the selected 3D blocks, the set of 2D patches belonging to each one of the images. Each set of 2D patches is denoised independently applying the collaborative strategy as proposed in [16].

**Fig 4:**
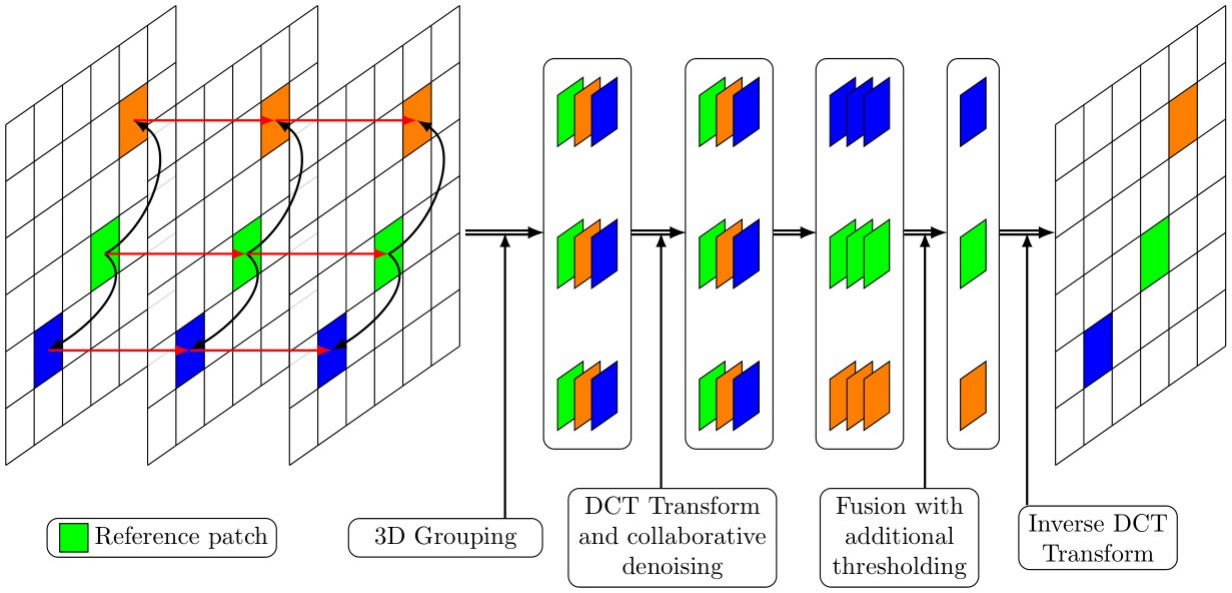
Processing scheme of a specific reference patch.

Since the fusion method described in Section 3 operates in the DCT domain, it can be integrated with the BM3D algorithm. For each group of 2D patches belonging to a particular exposure, a collaborative 2D+1D transform and threshold is applied. After applying the inverse 1D transform, and prior to the computation of the inverse 2D-DCT transforms, we group the 2D DCT coefficients of the patches belonging to the same selected spatial location and having different exposures. The multi-exposure fusion described by Eqs (1) and (3) is performed on each of these groups. After this fusion step, only one DCT transformed patch is obtained at each selected location, to which the inverse DCT is applied. Fig 4 illustrates the process.

By repeating the operation on each patch location and applying aggregation in the image domain, we obtain the final denoised and fused result. In our implementation no second iteration of the algorithm is performed.

## 5 Discussion and experimental results

In this section we compare the proposed method with state of the art algorithms for exposure fusion. We compare with Mertens et al. [1], Ma et al. [5], Li et al. [44], Kou et al. [28], Ma et al. [55], Hessel et al, (EEF) [19], Xu et al. [35], Zhang et al. (IFCNN) [64], Li et al. (CNNFEAT) [63], Hayat et al. (MEF-Sift) [22] and Martorell et al. [2]. The results from Ma et al. [5] and Ma et al. [55] were computed with the software downloaded from the corresponding author’s webpage. The results of Mertens et al. [1] were obtained from the dataset provided in [56, 72]. The code for Hessel et al. (EEF) [19, 73], Xu et al. (FusionDN) [35, 74], Zhang et al.(IFCNN) [64, 75], Li et al. (CNNFEAT) [63, 76] and Hayat et al. (MEF-Sift) [22, 77] were obtained from the corresponding GitHub webpages. The code by Xu et al., originally proposed to fuse only pairs of images, has been adapted to fuse any sequence. We fuse the first two images of the set, this output is then combined with the third input image, and so on until all the images in the sequence have been fused. The results from Li et al. [44], Kou et al. [28] and Martorell et al. [2] were computed with the code provided by the authors. In all cases, default parameter settings are adopted.

Our results were computed using the same parameters for all the tests in this section. We use patches of size 8 × 8 pixels which is the standard size used by patch based denoising algorithms, as for example the BM3D [16]. The threshold for the collaborative filtering is set to the standard value 2.7*σ*. For the fusion stage, we fixed *p* = 7 as the power exponent of the coefficient magnitudes in Eq (2), *σ*_*g*_ = 0.3 and *σ*_*l*_ = 0.5 for the combination of the *ξ* = 0 coefficients. These latter parameters were set experimentally.

A sliding window approach is applied for the DCT based denoising/fusion. Once it is processed, the window is moved along both directions with a displacement step of *N*_step_ = 2. The fact that the whole window is fused permits the processing of all the pixels in the image.

### 5.1 Noise-free sequences

In this case, we just compare the ability of fusing the different exposure images. We use for our method just the fusion algorithm described in section 3, but without applying any thresholding of the DCT. Note that none of the compared methods include this noise filtering step.

**Fig 5:**
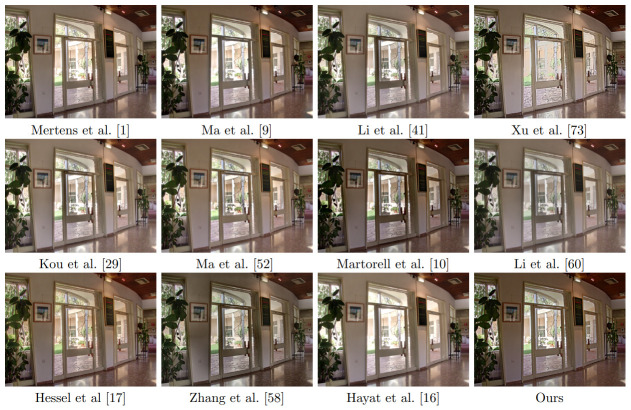
Results of fusion of noise-free multi-exposure images with different methods.

**Fig 6:**
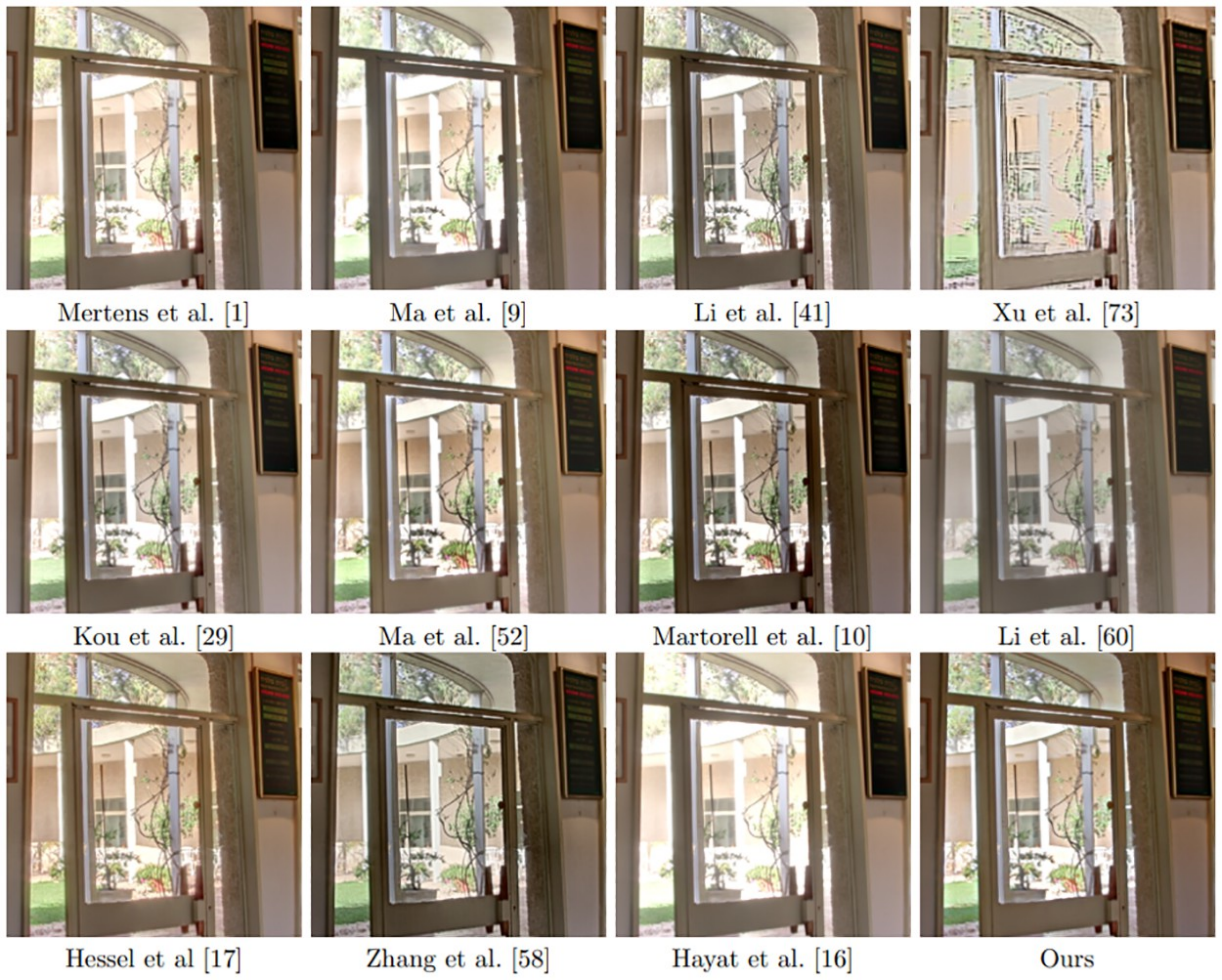
Excerpt of the results shown in Fig 5.

Fig 5 displays the results of all the methods on the “Belgium House” data set. Most methods produce a result with good global illumination. However, looking closer at the images in Fig 6, we observe that many outdoor details in Mertens et al. [1] and Ma et al. [5] are overexposed. Li et al. [44] and Kou et al. [28] are not able to maintain the letters on the blackboard on the right side of Fig 6 and Ma et al. [55] is not able to preserve the details on the tree at the top of the image. Li et al. [63] the fusion result is over-smoothed, while the result by Hayat et al. [22] is over-saturated at the bright parts. The results of [2], Hessel et al [19], Zhang et al. [64] and ours are quite similar.

### 5.2 Noisy sequences with white uniform Gaussian noise

**Fig 7:**
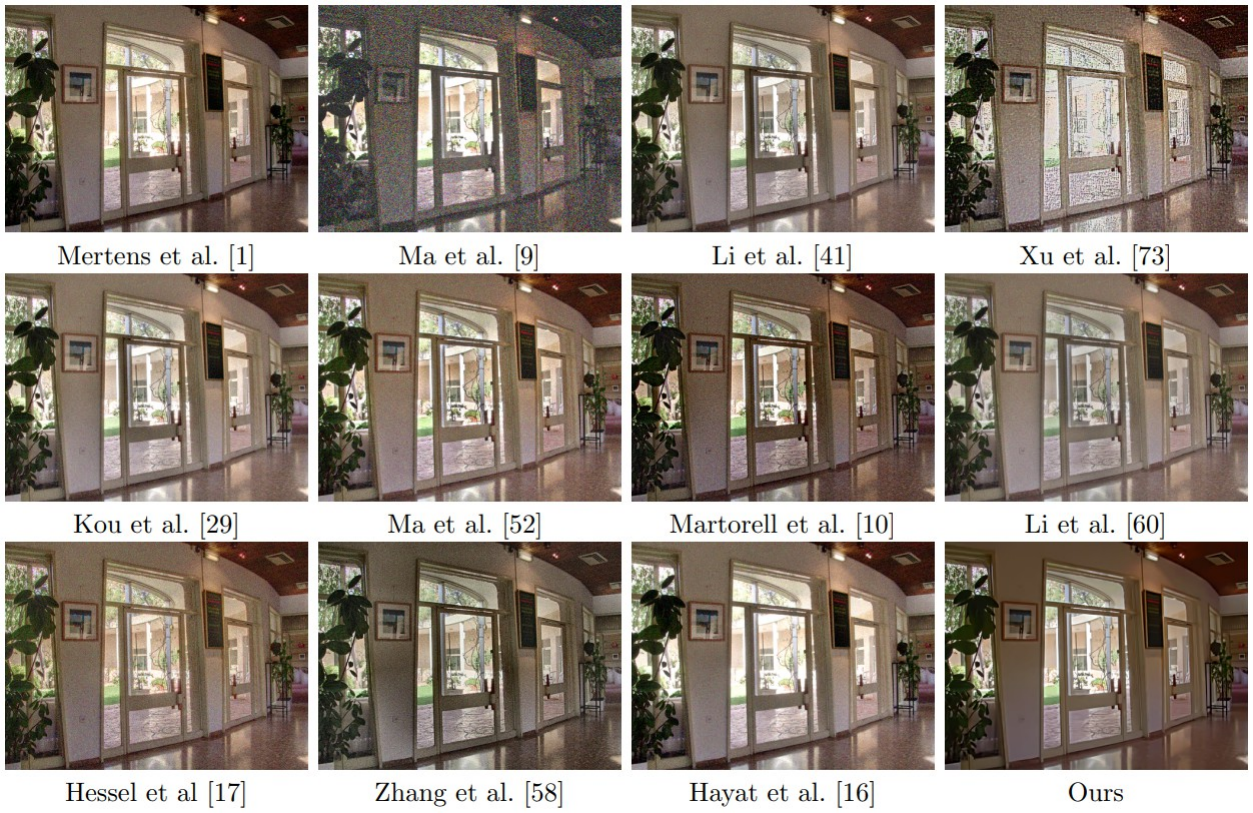
Results of fusion of noisy multi-exposure images with different methods. The noise standard deviation of each input image is 15.

**Fig 8:**
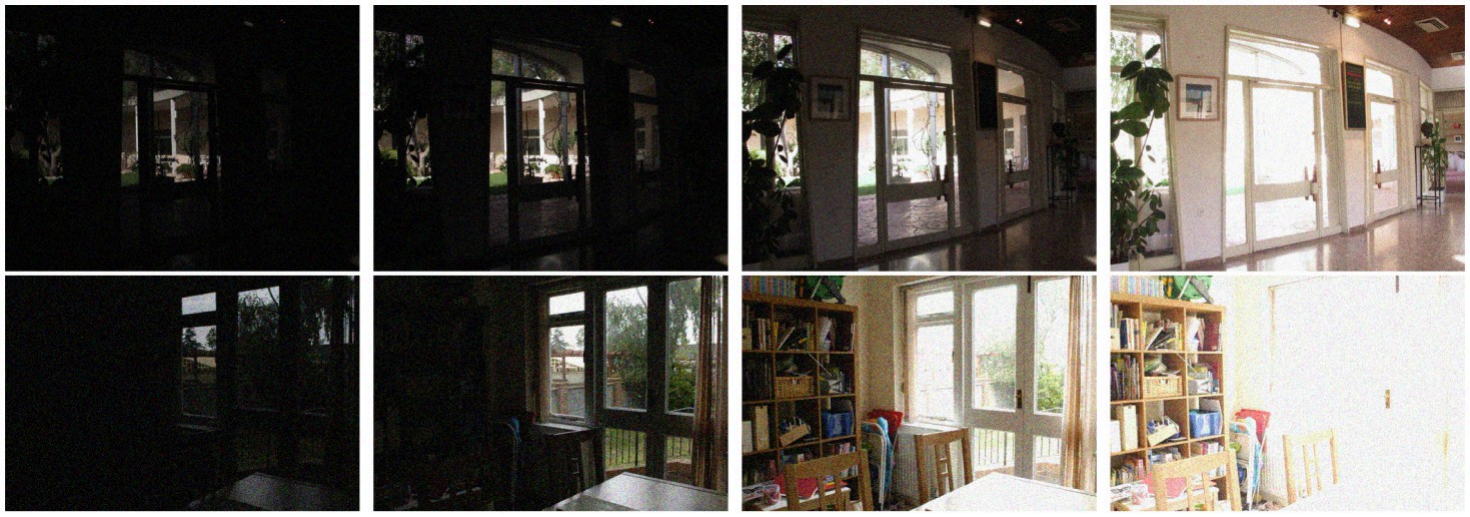
Noisy multi-exposure data sets used for comparison. On the first row, the noise standard deviation of each input image is 15. On the second row the standard deviation is 25.

**Fig 9:**
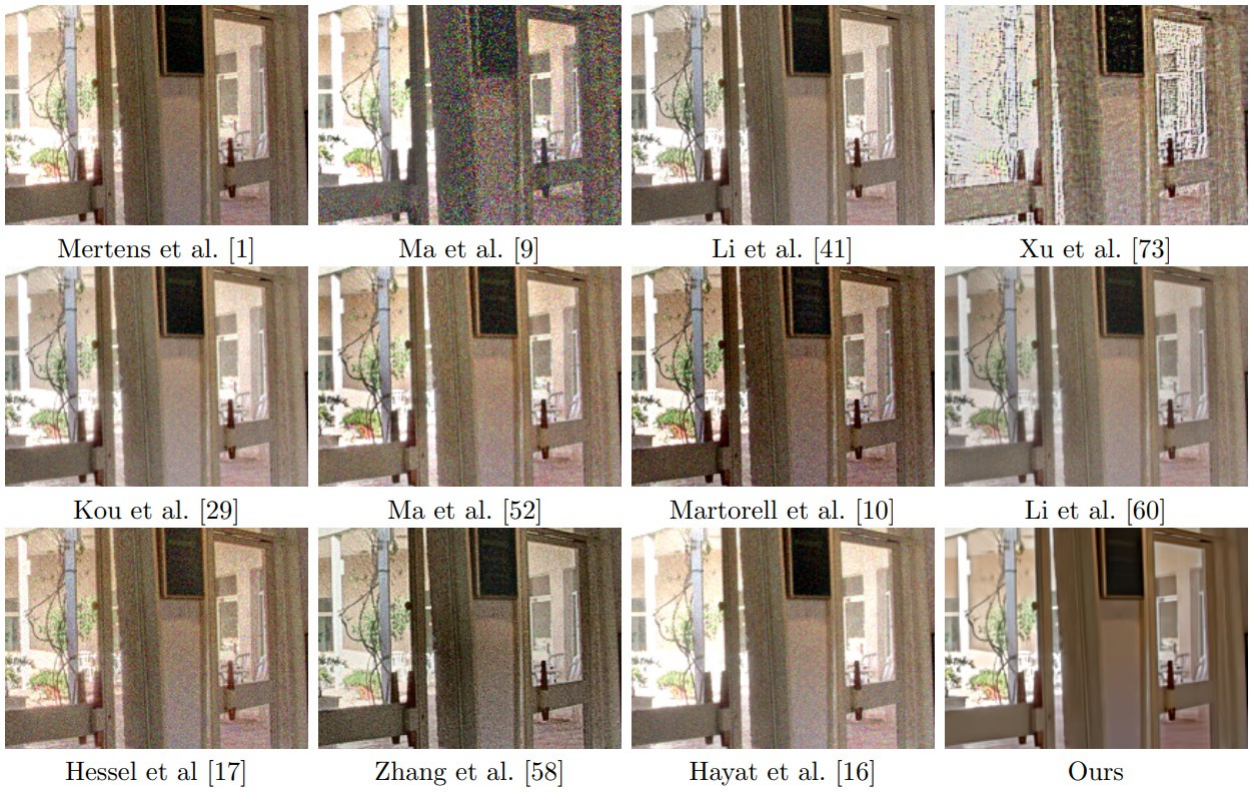
Excerpt of the results shown in Fig 8. It is clear from this figure, that our method is the only one that takes noise into account.

We compare in Fig 7 the results of all the algorithms on a noisy multi exposure sequence obtained after adding noise with standard deviation *σ* = 15 to the clean images (see Fig 8). We apply all the algorithms with their default parameters. It is clear from this figure, that none of the methods (except ours) is well adapted to the presence of noise. See Fig 9 for details of the results.

**Fig 10:**
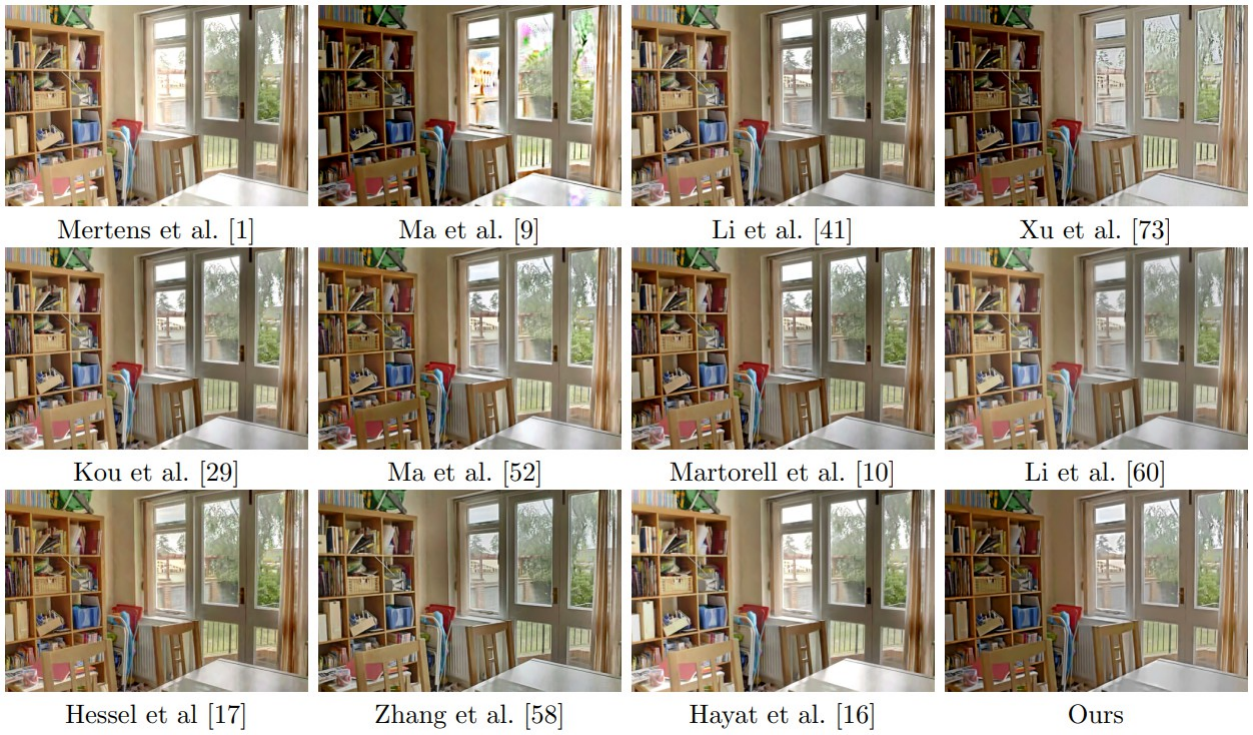
Results of fusion and denoising. Our method is the only one applied directly to the noisy multi-exposure images. The rest of methods fuse denoised versions of the images obtained using the BM3D algorithm. The noise standard deviation of each input image is 25.

**Fig 11:**
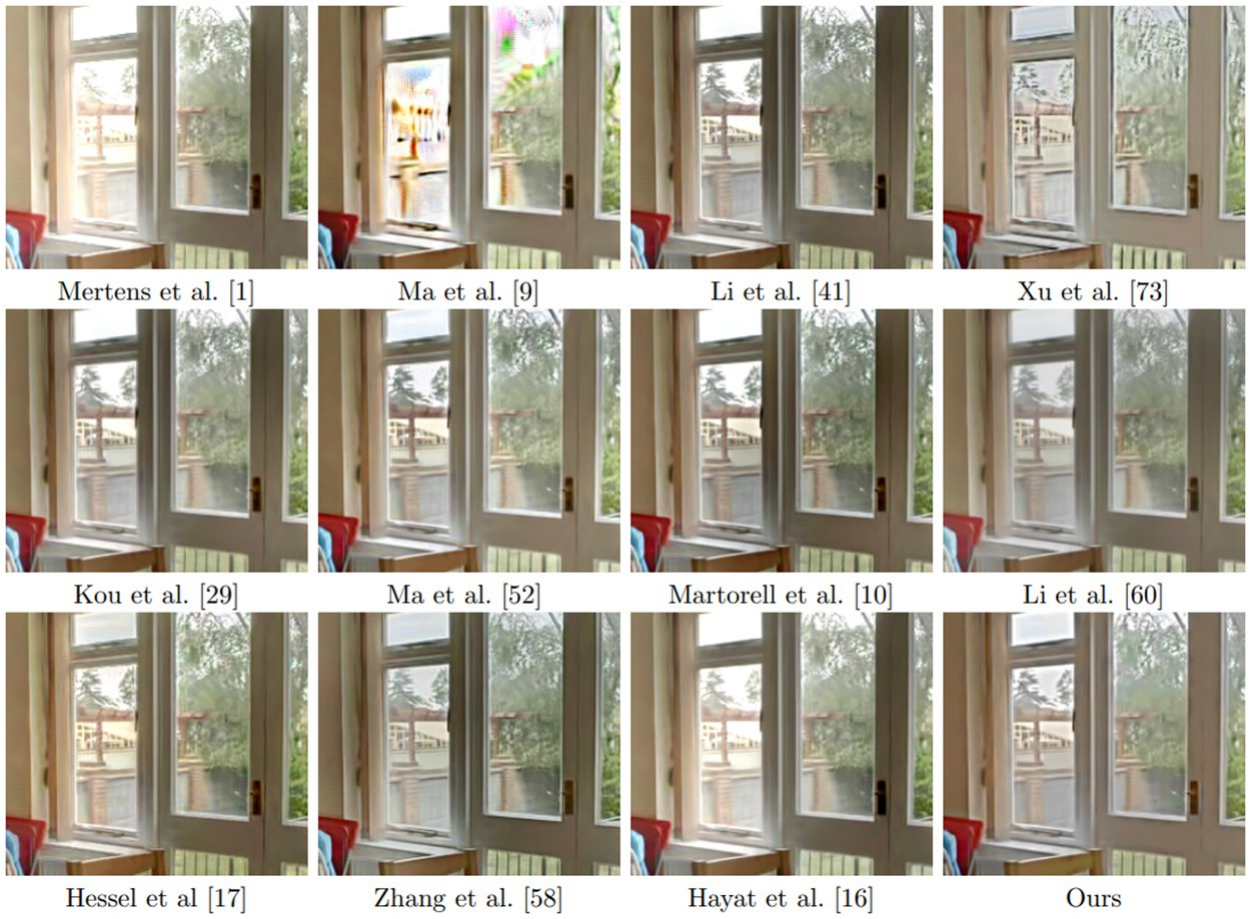
Excerpt of the results shown in Fig 10.

In the next experiment we add noise of standard deviation 25 to the clean multi-exposed images (see Fig 8) and apply the original BM3D algorithm [16] to denoise each one of them. We then apply the different multi exposure fusion methods to the denoised data (except our method, which is applied directly to the noisy sequence) and display the results in Fig 10. An excerpt of the results is zoomed in and displayed in Fig 11. We observe that our method is the only one able to denoise and fuse the multi exposure sequence without producing noticeable artifacts.

#### 5.2.1 Numerical evaluation

We propose a quantitative evaluation of the results by adapting the MEF-SSIM index proposed by Ma et al. [56] to take into account that the initial images are noisy.

We begin by briefly describing this measure. Ma et al. decompose each patch **x**_*k*_, *k* = 1, 2, …, *K* of the set of input images as

$$\begin{array}{c} x_{k}=\left|\right|x_{k}-\mu_{x_{k}}\left|\right|\cdot \frac{x_{k}-\mu_{x_{k}}}{\left|\right|x_{k}-\mu_{x_{k}}\left|\right|}+\mu_{x_{k}}=c_{k}\cdot s_{k}+l_{k} \end{array}$$ (8)

where $c_{k}=\left|\right|x_{k}-\mu_{x_{k}}\left|\right|$, $l_{k}=\mu_{x_{k}}$ and $s_{k}=\left(x_{k}-\mu_{x_{k}}\right)/\left|\right|x_{k}-\mu_{x_{k}}\left|\right|$ roughly represent the contrast, luminance and structure components of a patch **x**_*k*_. Using the previous decomposition, they compute the desired contrast and structure of the output image, respectively, as

$$\begin{array}{c} \hat{c}=\max_{k=1,\cdots ,K}c_{k}=\max_{k=1,\cdots ,K}\left|\right|x_{k}-\mu_{x_{k}}\left|\right|. \end{array}$$ (9)

and

$$\begin{array}{c} \bar{s}=\frac{\sum_{k=1}^{K}w\left(x_{k}-\mu_{x_{k}}\right)s_{k}}{\sum_{k=1}^{K}w\left(x_{k}-\mu_{x_{k}}\right)},\;\hat{s}=\frac{\bar{s}}{\left|\right|\bar{s}\left|\right|}, \end{array}$$ (10)

where *w*(⋅) is a weighting function that determines the contribution of each source image patch in the structure of the fused image patch (see Ma et al. [56] for more details on these weights.) With that, the desired output patch result is

$$\begin{array}{c} \hat{x}=\hat{c}\cdot \hat{s}. \end{array}$$ (11)

Finally, the value that measures the structural similarity between a set of input patches {**x**_*k*_}, *k* = 1, 2, …, *K* from a sequence of multi-exposure images and the corresponding patch of the fused image **y** is given by

$$\begin{array}{c} S\left(\left\{x_{k}\right\},y\right)=\frac{2\sigma_{\hat{x}y}+C}{\sigma_{\hat{x}}^{2}+\sigma_{y^{2}}+C}. \end{array}$$ (12)

The final measure is given by the mean of *S*({**x**_*k*_}, **y**) with {**x**_*k*_} centered at each pixel of the image

$$\begin{array}{c} Q\left(Y\right)=\frac{1}{M}\sum_{j=1}^{M}S\left(\left\{x_{k}\right\}\left(j\right),y\left(j\right)\right). \end{array}$$ (13)

The defined strength of the signal $\hat{c}$ needs to be modified in order to subtract the noise energy. Indeed, the strength of the noisy patches writes as the sum of the signal and noise energies. In order to evaluate the noise removal in the fused image **y**, we redefine

$$\begin{array}{c} \hat{c}=\sqrt{\max({\hat{c}}^{2}-\sigma^{2},0)}. \end{array}$$ (14)

We denote this modified measure as MEF-SSIM_n_. Larger values indicate a better performance of the method.

The values of MEF-SSIM_n_ for the examples of Figs 7 and 10 are displayed in Table 1. As it can be seen, our method has the best MEF-SSIM_n_ score.

**Table 1:** Values of MEF-SSIM_n_ for the examples of Figs 7 and 10.

	Mertens et al. [1]	Ma et al. [5]	Li et al. [44]	Xu et al. [35]	Kou et al. [28]	Ma et al. [55]	Martorell et al. [2]	Li et al. [63]	Hessel et al [19]	Zhang et al. [64]	Hayat et al. [22]	Ours
Fig 7	0.607	0.349	0.611	0.567	0.666	0.599	0.570	0.695	0.553	0.572	0.634	0.841
Fig 10	0.728	0.673	0.727	0.689	0.740	0.730	0.738	0.755	0.709	0.709	0.742	0.758

### 5.3 Realistic multi exposure noisy images

The AWGN model does not hold in practice for real photographs. The noise is approximately white and additive at the camera sensor. However, it is signal dependent, meaning that the noise standard deviation at each pixel depends on its noise-free value. The noise characteristics are then modified by the camera processing pipeline, consisting of demosaicking, color processing, gamma correction and compression [78].

In order to test our fusion and noise removal method with a ‘realistic’ noise case, we use RAW images which contain the acquired data at the sensor of the camera. This data can be obtained by selecting the RAW as output format when using professional reflex cameras. We add signal dependent white noise to a set of multi exposure RAW images and simulate a typical camera processing pipeline, obtaining their final color version in a common graphics format (PNG, JPEG, etc.).

**Fig 12:**
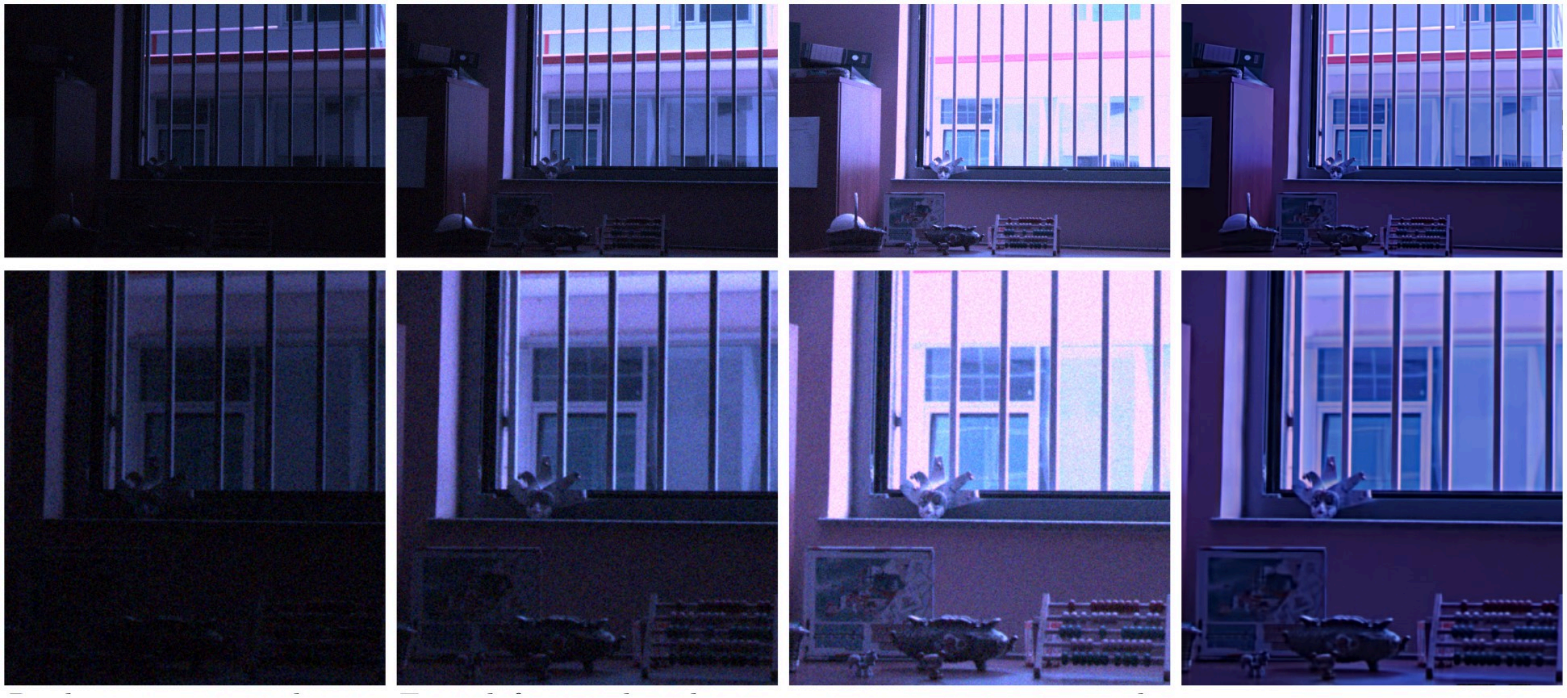
Realistic noise simulation. From left to right: three noisy images with different exposures and the fusion and noise removal result. Below an excerpt of each image.

The results in Fig 12 show that, even in this situation, the proposed method is able to denoise and fuse the images without introducing artifacts.

### 5.4 Computational analysis

The time complexity of the proposed algorithm is O(|Y|), where |*Y*| denotes the size of each image in the multi-exposure sequence.

Assuming that the 3D transforms used for the collaborative filtering are performed in a separable way (i.e. 2D transforms followed by 1D transforms, as detailed in [16]), the overall number of operations of the algorithm, per pixel, is approximately

$$\begin{array}{c} C_{T_{2D}}+2Kb^{2}N_{S}^{2}+2Kb^{2}C_{T_{1D}}+2Kkb^{2}+kC_{T_{2D}}^{\prime }+kb^{2} \end{array}$$ (15)

where:

- $C_{T_{2D}}$ denotes the number of operations required to compute the 2D DCT of the stack of patches similar to the one centered at the considered pixel. If we consider a neighborhood of size *N*_*S*_ × *N*_*S*_ around the pixel, this implies the computation of $KN_{S}^{2}$ 2D DCTs, where *K* is the number of images in the sequence. The time complexity can be reduced by pre-computing the transforms in each block of size *K* × *N*_*S*_ × *N*_*S*_ and reusing them in overlapping blocks, similarly to what is proposed in [16].
- The second term accounts for the 3D block matching step. This implies the exhaustive search, in a *N*_*S*_ × *N*_*S*_ neighborhood of the pixel, of 3D blocks of size *K* × *b* × *b*.
- The third term counts the number of operations for the computation of the 1D DCT transforms (direct and inverse) of the *k* nearest neighbors of each patch, in each frame. $C_{T_{1D}}$ denotes the cost of computing a 1D DCT transform (direct or inverse) of a vector of size *k*.
- The fourth term accounts for the fusion step, which involves *k* 3D blocks of patches of size *K* × *b* × *b*.
- The fifth term accounts for the number of operations needed to compute the *k* inverse 2D DCT transforms of the fused patches. $C_{T_{2D}}^{\prime }$ denotes the cost of computing the inverse 2D DCT transform of a patch of *b* × *b* pixels.
- Finally, the last term counts the number of operations involved in the image aggregation step.

Observe that the number of denoising operations per pixel, for each image, is smaller than that for the original BM3D algorithm, since only one step of the collaborative filtering is applied. In addition, the inverse DCT is applied only to the fused patches, since it is not necessary to denoise each individual image of the multi-exposure set.

Moreover, the previous estimation assumes that an exhaustive-search algorithm has been used for block matching. The costs $C_{T}$ of the DCT transforms depends on the availability of fast algorithms. By using predictive search techniques and fast separable transforms the complexity of the algorithm could be significantly reduced. Moreover, the overall number of operations can be further reduced by processing only one out of each *N*_step_ < *b* pixels in both the horizontal and vertical directions. Due to the overlapping of the patches, the aggregation step used in the final step of the algorithm guarantees that all the pixels are correctly processed. In this case, the overall complexity of the method is reduced by a factor $N_{\text{step}}^{2}$.

## 6 Conclusions

In this paper we propose a patch-based method for the simultaneous denoising and fusion of a sequence of multi-exposed images. Both tasks are performed in the DCT domain and take advantage of a collaborative 3D thesholding approach similar to BM3D [16] for denoising, and the proposed fusion technique. For the collaborative denoising, a spatio-temporal criterion is used to select similar patches along the sequence, following the approach in [8]. The overall strategy permits to denoise and fuse the set of images without the need of recovering each denoised image image in the multi-exposure set, leading to a very efficient procedure.

Several experiments show that the proposed method permits to obtain state-of-the-art fusion results even when the input images are noisy. As future work, we plan to extend the current approach to multi-exposed video sequences.
